# Vault differences in eyes implanted with spherical and toric implantable collamer lenses: an inter-eye analysis

**DOI:** 10.1186/s12886-022-02653-y

**Published:** 2022-11-15

**Authors:** Angel Sánchez Trancón, Santiago Cerpa Manito, Oscar Torrado Sierra, António Manuel Baptista, Pedro Miguel Serra

**Affiliations:** 1 Ophthalmology Clinic Vista Sánchez Trancón, Building Tecnolaser, Room 9, Calle La Violeta, 06005 Badajoz, Spain; 2 Centre of Physics, University of Minho, Braga, Portugal; 3 Optics and Optometry Department, Instituto Superior de Educação e Ciências, Lisbon, Portugal

**Keywords:** Implantable collamer lenses, Toric implantable collamer lenses, Vault, Efficacy index, Generalised estimating equations

## Abstract

**Purpose:**

To determine the influence of implantable collamer lenses (ICL) geometry, i.e. spherical and toric on the vault, and report the refractive and visual outcomes of patients bilaterally implanted with the two ICL geometries.

**Methods:**

This retrospective case series analysed 41 patients implanted with a spherical ICL (sICL) in one eye and an equal sized toric ICL (tICL) in the fellow eye. The anatomical and ICL-related parameters were assessed using anterior-segment optical coherence tomography (AS-OCT Visante, Zeiss Meditec AG) and optical tomography (Pentacam, OCULUS). The influence of the anatomical and ICL-related parameters on the vault was determined using generalised estimating equations (GEE) to incorporate inter-eye correlations.

**Results:**

Postoperative spherical equivalent was within ± 0.50D in 66% and 83% of the eyes, respectively implanted with sICL and tICL. The efficacy index in the sICL group was 1.06 and 1.14 in the tICL group. The mean inter-eye vault difference was -1.46 µm, anatomical and ICL-related parameters showed similar associations with the vault for sICL and tICL. The GEE identified the ICL size minus the anterior chamber width, the ICL spherical power and ICL central thickness as significant factors influencing the vault.

**Conclusions:**

Spherical and toric ICL showed good efficacy for the correction of myopia and astigmatism. Patients implanted bilaterally with sICL and tICL tend to present similar vaults. The vault produced by both types of ICL was mainly regulated by the oversizing of the ICL. This suggests that the ICL geometry (spherical vs toric) is a factor with limited influence on the vault, thus the sizing method of a sICL and tICL should be similar.

**Supplementary Information:**

The online version contains supplementary material available at 10.1186/s12886-022-02653-y.

## Introduction

Refractive surgery with implantation of phakic lenses such as Implantable Collamer Lenses (ICL, STAAR Surgical AG) has been shown to be a safe and efficient method for the correction of a wide range ametropias [1]. The clinical success of the surgery implies a good refractive outcome and the ability of the eye to maintain its physiological behaviour postoperatively. A relevant postoperative parameter contributing to the normal physiological behaviour regards to the distance between the ICL and the anterior surface of the crystalline lens, namely the vault [2]. The presence of high vaults may lead to the narrowing of the anterior chamber angle with potential influence on the intra-ocular pressure [3] while low vaults increase the chance of contact between the ICL and the crystalline lens which may interfere with crystalline lens epithelium cells metabolism [4]. The vault is regulated by a myriad of factors combining biometric and ICL-related parameters [5–9] added to the landing position of the ICL [10].

One of the parameters remaining under discussion regarding its influence on the vault is the ICL geometry, i.e. whether the ICL is spherical or toric. The two ICL modalities share the same sizes, optic zone diameters and haptics design but they differ in the optical zone geometry [11]. The spherical ICL (sICL) has a plano-concave geometry with a single radius of curvature in the posterior surface whereas the toric ICL (tICL) has a meridional variation of the curvature in the anterior surface to generate the astigmatic correction. Previous studies reported that tICLs produced higher vaults compared to the sICLs [12, 13]. However, these comparisons were done in independent groups of patients thus not controlling for variables known to influence the vault. Lege et al. suggested that differences in intraocular vault behaviour between tICL and sICL could be related to a higher stiffness of the former [14]. To address the limitations of previous studies, a recent study compared the vault of sICL and tICL when both ICLs were bilaterally implanted in the same patient [15]. The findings corroborate the dependence of the vault on a variety of biometric and ICL–related parameters, further adding that the cylinder power of the tICL was a contributing factor for the differences in the vault between sICL and tICL.

Since the predictability of the vault and inevitably the selection of the ICL size is a factor of the outmost relevance in ICL surgery, the investigation of whether the geometry of the ICL significantly contributes to the vault deserves more evidence. Thus, this study primarily aimed to determine the influence of ICL geometry on the vault, and secondly to report the refractive and visual outcomes achieved by patients bilaterally implanted with a sICL and tICL.

## Methods

### Study design

This retrospective case series comprised 41 patients who underwent uneventful, bilateral implantation of ICL with different geometry, a spherical and a toric ICL (EVO-V4c, STAAR Surgical AG, Nidau, Switzeland) between 2014 and 2017. The surgeries were performed on two separate days by two of the authors (SCM and AST), with the right eye being the first operated eye. The sample included patients with spherical refractive component between -3.00 and -20.00 D; astigmatic component lower than -5.00 D; internal anterior chamber depth (ACD), *i.e.*, distance (mm) between the corneal endothelium and the crystalline lens apex, ≥ 2.8 mm, endothelial cell density ≥ 2000 cells/mm^2^ and same ICL size in both eyes. Patients with ICL implanted vertically were excluded from the analysis. The ICL size and power were selected according to the manufacturer recommendations using the online calculator (OCOS™). The preoperative, postoperative and surgical protocol has been described elsewhere by our group [16]. This research followed the tenets of the Declaration of Helsinki and ethical approval was conceded by the local ethics committee (Comité Ético de Investigación Clínica de Badajoz). Patient informed consent was waived by the local ethics committee due to the retrospective nature of the study.

### Study parameters

**Fig. 1:**
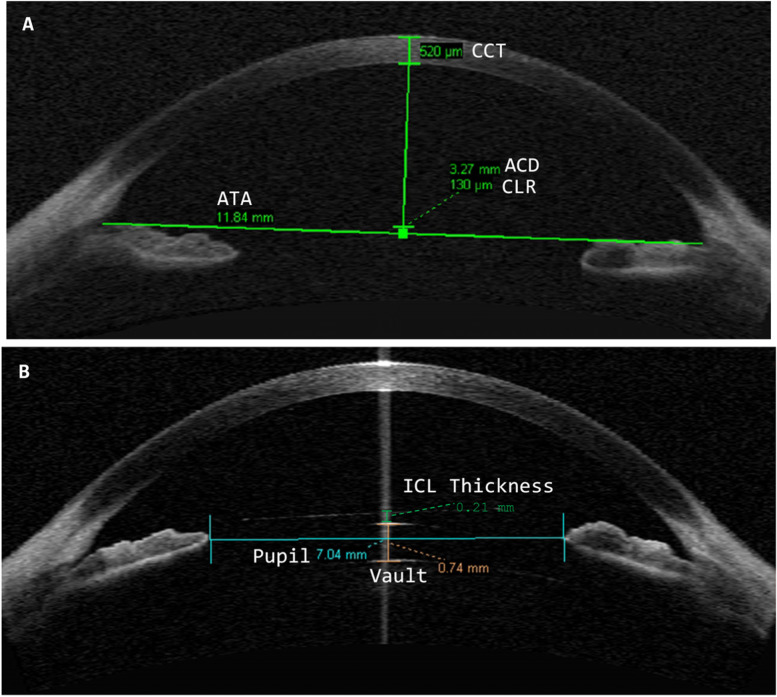
**A** Preoperative AS-OCT B-Scan used for measuring the horizontal anterior chamber distance (ATA), crystalline lens rise (CLR) and the internal anterior chamber depth (ACD) and central corneal thickness (CCT). **B** Postoperative AS-OCT B-Scan used for measuring the vault, the pupil diameter and the ICL thickness

Biometric preoperative parameters, namely white-to-white (WTW) i.e., the horizontal visible iris diameter (mm), simulated keratometry (Sim K), and central corneal thickness (CCT) were measured using optical tomography (Pentacam, OCULUS Optikgeräte, Wetzalar, Germany). Anterior segment optical coherence tomography (AS-OCT Visante, Zeiss Meditec AG, Jena, Germany) was used to detail the anterior segment anatomy by measuring the ACD, the horizontal anterior chamber distance (ATA) *i.e.*, the distance (mm) connecting the nasal and temporal iridocorneal angle recess, and crystalline lens rise (CLR) *i.e.*, the distance (µm) between the ATA line and the anterior surface crystalline lens apex, Fig. 1A. The CLR was regarded as positive if the crystalline apex was anterior to the ATA line and negative if posterior. The AS-OCT imaging was performed along the horizontal meridian using a single-scan centred on the pupil. Three months postoperatively the AS-OCT was used for measuring, the vault (µm) defined as the distance between the posterior ICL surface and the anterior surface crystalline lens apex; the pupil diameter (mm) as the distance between the nasal and temporal edge of the pupil and the ICL thickness as the distance (µm) between the anterior and posterior surface of the ICL in the thinnest part of the lens, Fig. 1B. Since the AS-OCT does not provide a quality centration index, all scans were checked by a proficient operator unaware of the modality of ICL implanted and measurements done using the device in-built callipers. The preoperative and postoperative anterior segment measurements were performed in both eyes prior to the instillation of diagnostic drugs in the same room with dim lighting conditions.

### Statistical analysis

The refractive, anatomical and ICL-related data from the eyes implanted with the sICL and tICL were reported with mean, standard deviation, range and 95% confidence interval for the mean. The predictability of the ICL refractive correction was analysed through the association between attempted and achieved spherical equivalent (SE); the refractive efficacy index was calculated as the ratio between the postoperative uncorrected distance visual acuity (UDVA) and the preoperative corrected distance visual acuity (CDVA). The preoperative and postoperative refractive data were reported in the spherical-cylinder form, with the cylinder in the negative corrective form. The tICL spherical-cylinder power was reported with the cylinder in the positive form. The tICL cylinder vectorial components J0 (Jackson-cross cylinder power at 180 and 90) and J45 (the Jackson-cross cylinder power at 45 and 135) were calculated as: J0 = -ICL Cylinder/2 × cos(2*ICL Cylinder axis) and J45 = -ICL Cylinder/2 × sin(2* ICL Cylinder axis), with the ICL Cylinder axis representing the axis orientation in the ICL cylinder positive form [17]. The normality of the pre and postoperative anatomical and ICL-related factors was assessed using the Kolmogorov–Smirnov test. Inter-eye comparisons for the different factors were performed using the paired t-test or Wilcoxon matched-pairs test, depending on the nature of the distribution. Bonferroni correction was used to account for the occurrence of type-I errors in multiple comparisons, the significance threshold was adjusted to 0.002 (0.05/22, where 22 corresponds to the total number of comparisons studied). An exploratory investigation of the influence of anatomical, refractive and ICL-related parameters on the vault was independently performed for sICL and tICL groups using bivariate linear regression analysis. Differences in the slopes magnitude were investigated through univariate analysis of variance, using the lens type (sICL or tICL) as fixed-factor. The effect of ICL size on the vault was investigated through multivariate analysis of variance (MANOVA). Finally, the association between vault (dependent variable) and the anatomical and ICL-related factors (independent variables), incorporating eyes implanted with sICL and tICL was assessed using generalised estimating equations (GEE), to account for the presence of inter-eye correlations [18]. The independent variables used in the GEE were selected based on the following criteria, (1) a single variable was chosen to describe a particular mechanism for the vault and (2) the variable chosen was the one presenting the highest association with the vault in the bivariate analysis. This study had a power of 0.951 to detect a difference of 80 µm (average of the differences between sICL and tICL reported in previous studies [12, 13, 15] and assuming a standard deviation of the differences equal to 120 µm [16]. Statistical analysis was performed using IBM SPSS Statistics V23.0.

## Results

The group mean age (mean ± SD) was 31.9 ± 8.1 years ranging from 21 to 50 years-old and comprised 23 (56.1%) women, Table 1. Patients had both eyes implanted with the same ICL size, 24 patients had the sICL implanted initially. The number of patients implanted with 12.6, 13.2 and 13.7 mm ICL were 9, 24 and 8, respectively.

**Table 1:** Preoperative and postoperative refractive and visual parameters for the eyes implanted with spherical (sICL) and toric ICL (tICL) and corresponding inter-eye differences. The values represent the mean ± standard deviation (SD), data range; 95% confidence interval for the mean. *p*-value for inter-eye comparisons (statistical significance after Bonferroni adjustment *p* ≤ 0.002)

**Parameter**		Eye Implanted with Spherical ICL	Eye Implanted with Toric ICL	Inter-eye comparisons	*p*-value
***Preoperative Manifest Refraction***					
Sphere (D)	Mean ± SD	-8.45 ± 3.08	-7.64 ± 2.96	-0.81 ± 2.40	0.037
	Range	-16.50; -2.50	-16.00; -3.00	-7.50; + 5.00	
	95% CI	-9.43; -7.64	-8.59; -6.70	-1.56; -0.06	
Cylinder (D)	Mean ± SD	-0.79 ± 0.43	-2.27 ± 0.63	+ 1.47 ± 0.70	< 0.0005*
	Range	-1.25; 0.00	-4.00; -1.50	+ 0.25; + 3.00	
	95% CI	-0.94; -0.67	-2.47; -2.07	+ 1.56; + 0.06	
Spherical Equivalent (D)	Mean ± SD	-8.85 ± 3.09	-8.77 ± 3.09	-0.08 ± 2.41	0.828
	Range	-17.00; -2.75	-17.50; -3.50	-6.63; + 5.63	
	95% CI	-9.84; -7.86	-9.73; -7.79	-0.84; + 0.68	
Preoperative CDVA (logMAR)	Mean ± SD	0.07 ± 0.08	0.11 ± 0.12	-0.03 ± 0.14	0.201
	Range	0.00; 0.30	0.00; 0.53	-0.48; + 0.26	
	95% CI	0.05; 0.10	0.07; 0.14	-0.08; + 0.01	
***Postoperative Manifest Refraction***					
Sphere (D)	Mean ± SD	+ 0.32 ± 0.55	+ 0.20 ± 0.44	+ 0.12 ± 0.58	0.208
	Range	-0.87; + 1.37	-1.12; -1.12	1.25; 0.00	
	95% CI	+ 0.15; + 0.50	-0.06; + 0.30	-0.69; + 0.51	
Cylinder (D)	Mean ± SD	-0.60 ± 0.28	-0.54 ± 0.39	+ 0.10 ± 0.56	0.246
	Range	-1.25; 0.0	-1.62; 0.0	-1.12; 1.50	
	95% CI	-0.68; -0.50	-0.66; -0.41	-0.28; + 0.07	
Spherical Equivalent (D)	Mean ± SD	+ 0.02 ± 0.53	-0.05 ± 0.44	+ 0.06 ± 0.57	0.474
	Range	-1.25; + 1.12	-1.37; + 0.87	-0.94; 2.06	
	95% CI	-0.15; + 0.19	-0.18; + 0.09	-0.12; + 0.25	
Postoperative UDVA (logMAR)	Mean ± SD	0.05 ± 0.07	0.06 ± 0.08	-0.01 ± 0.01	0.566
	Range	-0.08; 0.22	0.00; 0.22	-0.30; + 0.22	
	95% CI	0.03; 0.07	0.03; 0.09	-0.04; + 0.02	
Efficacy Index	Mean ± SD	1.06 ± 0.14	1.12 ± 0.20	-0.06 ± 0.26	0.091
	Range	0.80; 1.50	0.78; 1.67	-0.67; + 0.61	
	95% CI	1.01; 1.11	1.01; 1.19	-0.14; + 0.02	

^*^Statistical significance

### Predictability and efficacy

**Fig. 2:**
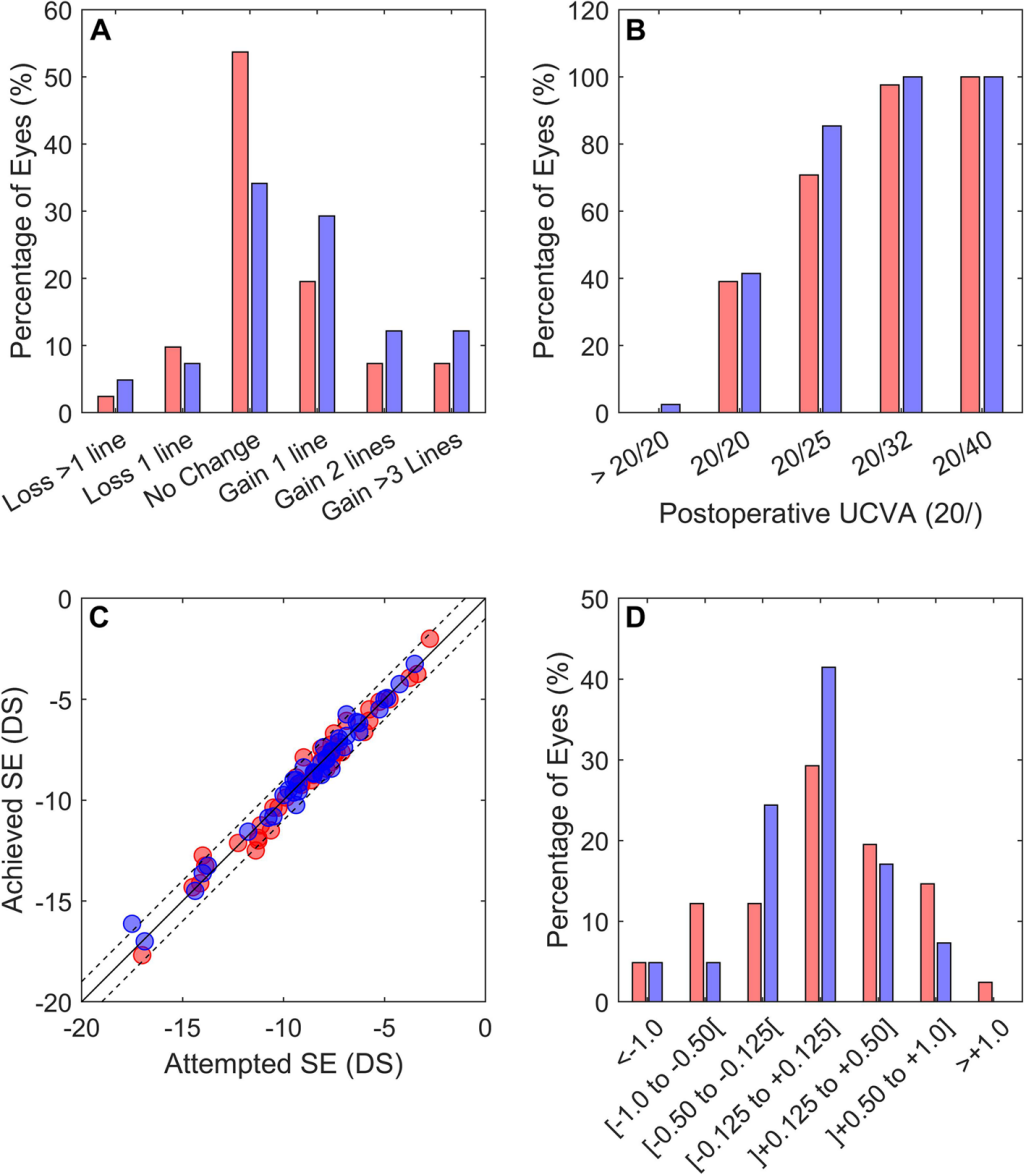
Refractive outcomes where red stands for Spherical ICL (sICL) and blue for Toric ICL (tICL). **A** Postoperative Uncorrected Distance Visual Acuity (UCVA) improvement from preoperative corrected distance VA (CDVA); **B** Cumulative postoperative Snellen (imperial) UCVA; **C** Predictability, association between Attempted SE and Achieved SE, dashed lines represent ± 0.50D from the equity line; **D** Postoperative refractive error distribution

The eyes implanted with a sICL had an efficacy index (Postoperative UDVA/Preoperative CDVA) of 1.06. Approximately 12% of the eyes (*n* = 5) lost one or more VA lines, 54% had no change in VA (*n* = 22) and 34% (*n* = 14) improved VA by one or more lines, Fig. 2A. All eyes with sICL had an UCVA better or equal than 20/40 and 70.7% had an UCVA better than 20/25, Fig. 2B. Nearly 66% of the eyes (*n* = 27) had a postoperative manifest SE within ± 0.50D and 92.7% (*n* = 38) within ± 1.00D, Fig. 2C and D. Regarding the postoperative refractive cylinder 19.5% (*n* = 8) of the eyes had a residual cylinder lower than 0.25D and 48.8% (*n* = 20) lower than 0.50D.

The fellow eyes implanted with tICL had an efficacy index of 1.12. Nearly 12% of these eyes (*n* = 5) lost one or more VA lines, 34.1% showed no improvement in VA and 53.6% improved VA by one or more lines, Fig. 2A. All eyes with tICL had postoperative UCVA better or equal than 20/40 and 80.5% had an UCVA better than 20/25, Fig. 2B. Approximately 82.9% (*n* = 34) of the eyes implanted with tICL had a postoperative manifest SE within ± 0.50D (*n* = 34) and 95.1% (*n* = 39) within ± 1.00D, Fig. 2C and D. The postoperative refractive cylinder was lower than 0.25D in 31.7% (*n* = 13) and lower than 0.50D in 63.4% (*n* = 26) of the eyes.

### Anatomical and ICL-related parameters

Comparison between the following preoperative anatomical parameters, ATA, WTW, ACD, CLR, CCT and Sim K, showed no statistically significant differences between fellow eyes (*p* > 0.153 for all), Table 2. Corneal astigmatism was higher in the eyes implanted with tICL (difference: -0.82 D, *p* < 0.0001). Regarding the ICL-related parameters, the tICL group had on average an ICL with higher spherical component (difference: -0.91 ± 2.89 D), however the difference was not statistically significant. Both eyes had implanted ICLs with the same difference between ICL size and ATA (*p* = 0.799) and ICL with similar central thicknesses (*p* = 0.126).

**Table 2:** Preoperative and postoperative anatomical and ICL-related parameters of the eyes implanted with spherical (sICL) and toric ICL (tICL); and corresponding inter-eye differences. The values are represented by the mean ± standard deviation (SD), data range and the 95% CI for the mean. *p*-value for inter-eye comparisons (statistical significance after Bonferroni adjustment *p* ≤ 0.002)

**Parameter**		**Eye Implanted with Spherical ICL**	**Eye Implanted with Toric ICL**	**Inter-eye Difference**	*p*-value
ATA (mm)	MD ± SD	12.21 ± 0.51	12.20 ± 0.49	0.01 ± 0.49	0.799
	Range	10.72; 13.48	11.01; 13.56	-0.31; 0.62	
	95% CI	12.05; 12.37	12.05; 12.37	-0.05; 0.07	
WTW (mm)	MD ± SD	11.8 ± 0.5	11.76 ± 0.55	0.0 ± 0.3	0.465
	Range	10.7; 12.8	10.00; 12.90	-0.2; 1.8	
	95% CI	11.6; 11.9	11.6; 11.9	-0.1; 0.1	
ICL size-ATA (mm)	MD ± SD	0.96 ± 0.37	0.96 ± 0.32	0.01 ± 0.49	0.799
	Range	0.22; 1.88	0.14; 1.59	-0.31; 0.62	
	95% CI	0.84; 1.07	0.86;1.06	-0.05; 0.07	
ICL size-WTW (mm)	MD ± SD	1.4 ± 0.3	1.4 ± 0.4	0.0 ± 0.3	0.465
	Range	0.8; 2.0	0.6; 2.2	-1.8; 0.2	
	95% CI	1.3; 1.5	1.3; 1.5	-0.1; 0.1	
ACD (mm)	MD ± SD	3.28 ± 0.24	3.29 ± 0.23	-0.01 ± 0.49	0.432
	Range	2.80; 3.90	2.82; 3.84	-0.32; 0.27	
	95% CI	3.20; 3.35	3.12; 3.36	-0.04; 0.02	
CLR (µm)	MD ± SD	+ 136.5 ± 207.1	+ 114.1 ± 210.5	22.4 ± 0.49	0.153
	Range	-330.0; + 730.0	-350.0; + 710.0	-220.0; 220.0	
	95% CI	+ 71.2; + 202.0	+ 47.7; + 180.6	-8.7; 53.6	
CCT (mm)	MD ± SD	527.8 ± 35.2	529.8 ± 41.2	2.0 ± 16.0	0.440
	Range	459.5; 596.0	449.9; 609.5	-60.0; 20.0	
	95% CI	516.7; 538.9	516.7; 542.8	-7.0; 3.1	
Sim K (D)	MD ± SD	43.9 ± 1.7	43.8 ± 1.7	0.6 ± 0.3	0.290
	Range	40.0; 47.8	40.0; 47.9	-0.5; 0.8	
	95% CI	43.3; 44.4	43.2; 44.4	-0.1; 0.2	
Corneal Astigmatism (D)	MD ± SD	+ 1.08 ± 0.47	+ 1.90 ± 0.60	0.8 ± 0.3	< 0.0001*
	Range	0.16; 1.98	0.73; 3.06	-1.0; -0.6	
	95% CI	0.93; 1.22	1.71; 2.09	-2.2; 0.3	
ICL Sphere (D)	MD ± SD	-9.88 ± 2.91	-10.79 ± 2.86	0.91 ± 2.89	0.014
	Range	-17.00; -3.00	-18.00; -5.00	-5.00; 5.50	
	95% CI	-10.81; -8.95	-11.71; -9.88	0.19; 1.63	
ICL Cylinder (D)	MD ± SD	-	+ 2.21 ± 0.64	-	-
	Range		+ 3.50; + 1.50		
	95% CI		+ 2.00; 2.40		
ICL J0 Cylinder (D)	MD ± SD	-	0.47 ± 0.83	-	-
	Range		-1.25; + 1.72		
	95% CI		0.20; 0.73		
ICL J45Cylinder (D)	MD ± SD	-	-0.09 ± 0.64	-	-
	Range		-1.00; + 1.50		
	95% CI		-0.29; 0.12		
ICL Thickness (µm)	MD ± SD	205.8 ± 18.9	201.1 ± 21.1	4.7 ± 19.1	0.126
	Range	160.0; 230.0	150.0; 250.0	-30.0; 70.0	
	95% CI	199.8; 211.8	194.5; 207.8	-1.4; 10.7	
Vault (µm)	MD ± SD	624.6 ± 273.8	626.1 ± 254.5	-1.5 ± 143.4	0.949
	Range	120.0; 1140.0	90.0; 1150.0	-360.0; 350.0	
	95% CI	538.2; 711.1	545.8; 706.4	-47.4; 44.4	
Pupil (mm)	MD ± SD	5.6 ± 1.0	5.4 ± 1.1	0.2 ± 0.5	0.022
	Range	2.9; 7.9	3.2; 8.1	-0.9; 1.6	
	95% CI	5.2; 5.9	5.0; 5.7	0.0; 0.4	

^*^Statistical significance *p* ≤ 0.002

Postoperatively, the eyes implanted with the sICL and tICL had similar vaults and pupil sizes. The mean vault difference (sICL–tICL) between fellow eyes was -1.5 ± 143.4 µm.

### Association between vault, anatomical and ICL-related parameters

**Fig. 3:**
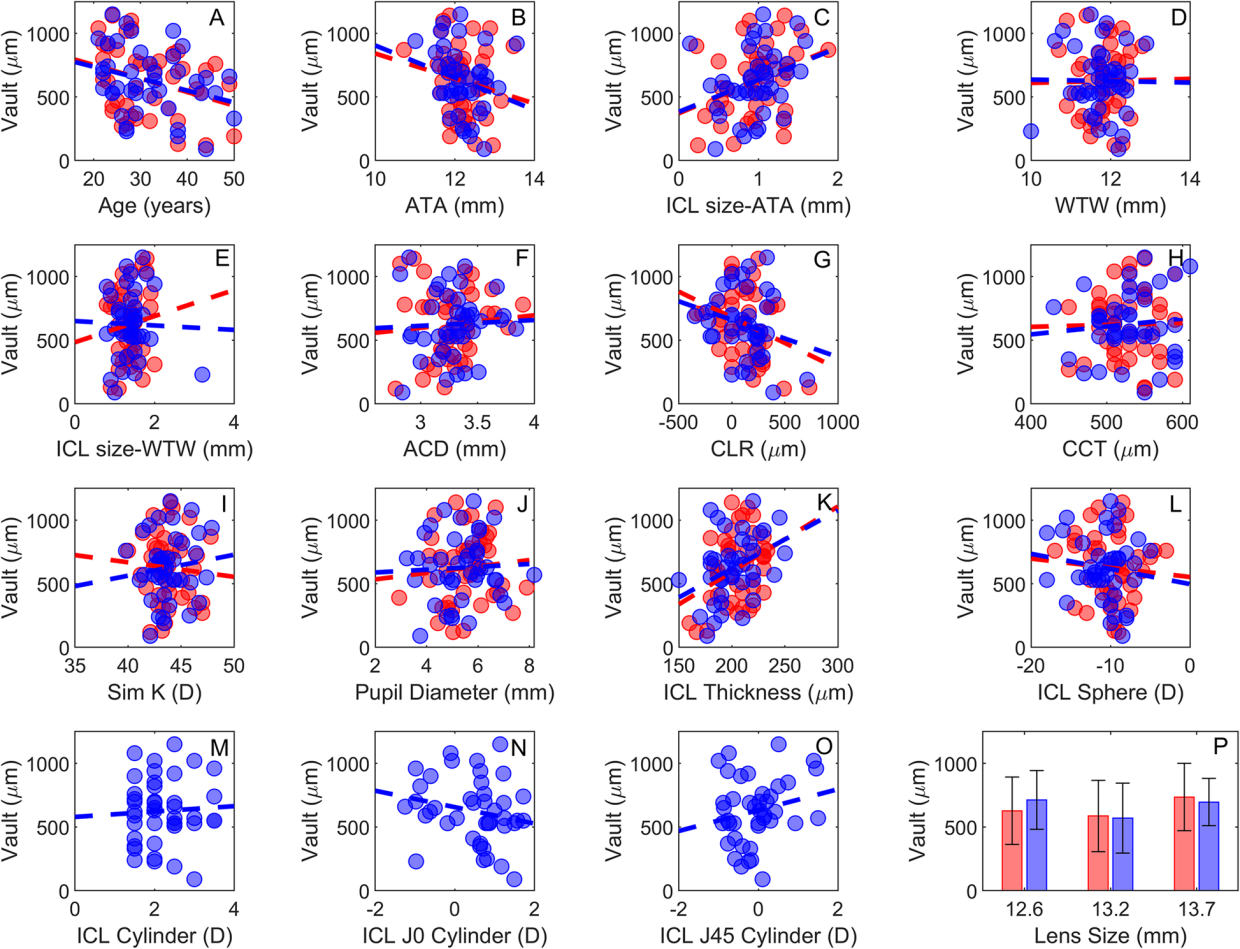
Association between vault and independent variables, where red stands for Spherical ICL and blue for Toric ICL. **A**-Age, **B**- ATA, **C**- ICL size minus ATA, **D**- WTW, **E**- ICL size minus WTW, **F**- ACD, **G**- CLR, **H**- CCT, **I**- Sim K, **J**- Pupil Diameter, **K**- ICL Thickness, **L**- ICL Sphere, **M**- Cylinder, **N**- ICL J0 Cylinder, **O** – ICL J45 Cylinder, **P**-Lens size

Individual bivariate correlation analysis showed that the vault in the eyes implanted with sICL was negatively correlated with the patient age (*R* = -0.31, *p* = 0.046), positively correlated with the ICL size minus the ATA (*R* = 0.36, *p* = 0.021); and positively correlated with the ICL central thickness (*R* = 0.35, *p* = 0.023). The vault in eyes implanted with tICL was correlated with the ICL size minus the ATA (*R* = 0.36, *p* = 0.021) and ICL thickness (*R *= 0.38, *p* = 0.014), Fig. 3 and Supplementary Table S1. All the remaining parameters yielded no statistical significant associations with the vault (*p* < 0.05). There were no statistical differences between slopes (variation of the vault with the independent variable) corresponding to the two types of ICL (*p* >  > 0.05), Fig. 3 A-L, informing that anatomical and ICL-related-factors have a similar influence in both type of lenses concerning the vault. Regarding the ICL size, the vaults produced by the three ICL sizes were similar in the sICL (*p* = 0.336) and tICL (*p* = 0.286) groups, Fig. 3 (P). Furthermore, the inter-eye vault differences were -57.0 ± 118.4 µm, 1.7 ± 140.7 µm and 24.3 ± 135.7 µm, respectively for the 12.6, 13.2 and 13.7, however without statistically significant differences (*p* = 0.327).

Combining the eyes implanted with the sICL and tICL and accounting for the inter-eye associations, the GEE indicated that the vault was associated with the ICL size minus the ATA (*p* < 0.0001), ICL sphere component (*p* = 0.013), with ICL thickness (*p* = 0.016), Table 3. Neither the type of lens (sICL or tICL) (Wald χ^2^ = 0.354 *p* = 0.552) or ICL size (12.6, 13.2 and 13.7 mm) (Wald χ^2^ = 2.25 *p* = 0.325) were significantly associated with the vault.

**Table 3:** Generalised estimation equation parameters, B- unstandardized coefficient, standard error, 95% confidence interval limits and significance. ICL type and ICL size were defined as categorical variables

**Predictors**	**Coefficients**				
	B	Standard Error	95% Confidence Interval		Sig
***Constant***	791.39	994.13	-1157.08	2739.86	0.426
***ICL Type***					
sICL (reference)	0.00	-	-	-	-
tICL	5.27	40.40	-73.91	84.46	0.552
***ICL Size (mm)***					
12.6 (reference)	0.00	-	-	-	-
13.2	-135.95	101.83	-335.53	63.62	0.182
13.7	-52.28	128.82	-304.69	200.12	0.685
Age (years)	-7.12	3.81	-14.59	0.348	0.062
ICL size – ATA (mm)	386.53	87.027	215.96	557.10	< 0.0001*
ACD (mm)	39.33	174.30	-302.28	380.95	0.603
CLR (mm)	-0.09	0.16	-0.43	0.14	0.279
CCT (µm)	1.07	0.92	-0.73	2.86	0.245
Sim K (D)	-38.31	20.21	-77.91	1.29	0.058
Pupil (mm)	-3.54	22.44	-47.52	40.4	0.875
ICL Thickness (µm)	2.84	1.33	0.60	5.83	0.034*
ICL Sphere (D)	-21.96	8.76	-39.13	-4.79	0.012*
ICL J0 Cylinder vector (D)	-46.18	37.98	-120.63	28.27	0.224
ICL J45 Cylinder vector (D)	-5.14	61.75	-126.16	115.89	0.934

*Statistical significance

## Discussion

Up to date, the mechanisms influencing the vault can be divided to those associated to the anatomic features of the eye [2, 6, 8, 9, 19–22], the landing position of the ICL [10] and those related to ICL physical parameters [9, 23, 24]. This later group comprises the ICL dioptric power [9, 23] and the ICL size [9, 24]. Another factor, with potential influence on the vault is the optical geometry of the ICL, i.e. whether the ICL is spherical or toric [12–15]. In the present study, sICL and tICL showed no difference in vault, and the associations of the vault with anatomical and ICL-related parameters were similar for both ICL types. Also, the refractive outcomes showed good predictability and effectiveness of ICL in correcting a wide range of myopia and moderate levels of astigmatism.

The results point towards a good predictability of sICL and tICL surgery, respectively with 66% and 83% of the eyes presenting a postoperative SE within ± 0.50 D; 93% and 95% showed a postoperative SE within ± 1.00 D. Additionally, the tICL group showed a higher percentage of eyes with lower levels (≤ 0.50 D) of postoperative astigmatism 63.4% compared to 48.8% in the sICL group. This contributed to an improvement of one or more VA lines in 34% and 54%, respectively in the eyes implanted with sICL and tICL resulting in a higher efficacy of the tICL. Garcia del la Rosa et al. in a group of patients implanted with sICL and tICL (ICL-V4b) reported at 12-months follow-up a SE within ± 0.50 D in 85% of the eyes, with approximately 45% of the eyes improving one or more VA lines [25]. Niu et al. reported in a group of high myopes (preoperative SE:-14.00 D) implanted with sICL and tICL, a postoperative SE within ± 0.50D in 73% of the eyes and within ± 1.00 D in 80%, however some of their eyes had myopia above the ICL correction limit which increases the postoperative SE [26]. Moshirfar et al. reported at 3-months follow-up in a group of eyes implanted with tICL a postoperative SE within ± 0.50D in 77% of the eyes and a reduction in refractive astigmatism from 2.67 D to 0.68 D, which concurs with the present study [27]. Zhao et al. in a prospective study of patients bilaterally implanted with sICL and tICL reported a postoperative SE within ± 0.50 D in 94% and 88% of the eyes implanted with sICL and tICL, respectively, representing lower levels of postoperative refractive error compared to the present findings [15]. The higher refractive predictability in Zhao’s study may be related to the cylinder power threshold used for tICL implantation (present study: 1.25 D; Zhao et al.: 0.75 D). This resulted in 40% (*n* = 17) of the eyes with postoperative astigmatism ≥ 0.75 D compared to none in Zhao’s et al. study. These outcomes point towards the importance of considering tICL in the correction of low refractive astigmatism, since it influences the visual performance [28] and the subjective quality of vision [29].

Regarding the vault differences between the two ICL types, there was no clinically significant inter-eye difference (sICL-tICL: -1.5 ± 143.4 µm). Also, both ICLs geometries showed similar relationships between vault magnitude and anatomical or ICL-related parameters. The present results do not show evidence of tICL parameters such as the cylinder magnitude or vectorial components on the vault, as observed by correlation analysis in Fig. 3 M–O. This evidence is corroborated when the association of all the independent parameters studied with the vault is investigated by merging the sICL and tICL groups though the GEE. This finding contrasts with a recent study using a similar inter-eye analysis [15]. Zhao et al. reported an average tICL vault 110 µm higher than the produced by sICL, with the cylinder power being the contributing factor for the difference in the vault between the two ICL geometries. Their estimated ICL cylinder contribution corresponded to an increase of 78 µm in vault per dioptre of cylinder. Previous studies using the ICL-V4b model reported smaller differences between sICL and tICL, 90 µm [12] and 45 µm [13] when the two types of ICL were implanted in different groups of patients. Alfonso et al. stated that the difference in vault was associated to the toricity in the posterior surface of the ICL, thus the lower radius of curvature necessary to produce the cylinder component increased the sagittal depth in the optic zone [13]. Alternatively, Lege et al. suggested that differences in the intraocular behaviour between sICL and tICL during accommodation were related to the higher rigidity of the tICL [14]. The ICL model used in this study (ICL-V4c) has the toric tailored on the anterior surface (STAAR Spain personal communication), presumably as a convex surface (positive cylinder). Thus, the hypothetical influence of tICL geometry on the vault may be attributed to differences in the ICL thickness across the meridians, produced by the presence of meridional curvature variations in the ICL anterior surface, which in turn changes the mechanical properties of the ICL. However, the present results do not show significant influence of tICL geometry on the vault, suggesting that ICL geometry plays a minor role in vault magnitude.

As far as the main vault predictors are concerned, ICL size minus the ATA represents the most influential predictor, characterizing the level compression of the ICL due to its oversize. Using the ATA as a descriptor of the anterior chamber width, the GEE model predicted a variation of 387 µm in vault per millimetre of compression, with this prediction applicable to the two ICL geometries. The present finding concurs with Igarashi et al. results in the sense that the ICL size minus the ATA could be used as a single predictor of the vault, with a variation of 661 µm in vault per millimetre of compression [21]. Similar findings were reported by Sánchez-Trancón et al., with the vault variation comprised between 318 µm to 528 µm per millimetres of compression depending on the ICL size [9]. On the other hand, we did not find a significant association between vault and the compression calculated using the ICL size minus WTW. This result agrees with previous studies that found a weaker association between the vault and the compression calculated using the WTW compared to compression calculated using the ATA [9, 21] or using the sulcus-to-sulcus (STS) distance [5]. Accounting for this, is the fact that the ICL haptics rest on the ciliary-sulcus complex, thus the STS represents the most realistic measurement to calculate the ICL compression. The anterior chamber width (e.g. ATA) has been shown to have a stronger association with the STS compared to the WTW [30, 31]. These findings support the argument that the anterior chamber width can be used as an anatomical parameter for improving the ICL manufacturer’s sizing nomogram.

A second relevant vault predictor was the ICL spherical power. The GEE estimated an increment of about 20 µm in vault for every negative unit of spherical dioptre, which is in agreement with the SE association reported by Sánchez-Trancón et al. in a larger sample [9] and similar to the 27 µm reported by Hernandez-Matamoros [23]. This reflects the increase in the innate sagittal depth of an ICL, related to a more pronounced concave ICL posterior surface radius as the spherical power becomes more myopic. Lee et al. reported an innate vault variation of about 45 µm per dioptre for ICLs (model: ICM-V4) ranging from -3.0 to -23.0 D [5]. Thus, when estimating the vault of an ICL the spherical power should be considered since more myopic ICLs tend to originate higher vaults whereas, less myopic ICL produce lower vaults. For the tICL the sphere component (with the cylinder in positive form) should be considered since the intrinsic vault of the ICL depends on the spherical power of the most negative meridian.

A third vault predictor was the ICL thickness, with the GEE predicting an increase of about 2.8 µm in the vault per one µm of increase in the ICL central thickness. Considering the full range of thicknesses measured (~ 100 µm) the maximum vault difference estimated would be 280 µm. A possible explanation for the thickness influence on the vault is that thicker ICLs are stiffer and be less likely to be influenced by the iris compression forces. Regarding the two ICL geometries, the central thickness in the sICL and tIC were proximal and both had similar relationships with the vault. The fact that the central ICL thickness could not be associated to any other ICL-related parameter, for instance ICL sphere (sICL: *R* = 0.08 *p* = 0.631; tICL: *R* = -0.18, p = 0.270, data not shown) limits the use of ICL central thickness as a predictor of the vault. Considering the peripheral ICL thickness may result in a better vault predictor, as the ICL peripheral thickness increases with spherical power while the central thickness tends to remain constant [32]. Future studies are required for detailing the variation of the ICL thickness in the central and peripheral parts of the ICL and study its influence on the vault. Meanwhile, the theoretical assumptions that more myopic ICLs have thicker peripheral parts and tICL with higher cylinders have thicker central parts, may guide in understanding the effect of ICL thickness on the vault.

Other parameters representative of additional mechanism regulating the vault such as the CLR [7–9, 22, 24, 33], age [9, 19], ICL size [9, 24] and pupil size [32, 34–36] failed to show statistical significance in the GEE model. In the bivariate analysis, patient age showed some degree of association (negative) with the vault, which can be attributed to two age-related factors, the increase in crystalline lens anterior protrusion (CLR) [22] and the decrease in pupil diameter [37]. The former reduces the vault created by the ICL compression and its intrinsic vault, and the later increases the anterior–posterior pressure produced by the iris placing the ICL closer to the crystalline. The vault showed a negative association with the CLR for both ICL modalities, indicating that crystalline lens morphology plays a role in vault magnitude [8, 33] however the correlations did not reach statistical significance. Gonzalez-Lopez et al. reported that eyes with high vault (> 750 µm) had an average CLR of + 73 µm whereas eyes presenting low vault (< 100 µm) had an average CLR of + 350 µm [22]. Cerpa et al. further suggested that eyes with CLR >  + 150 µm were at risk of presenting low vault (< 250 µm) [24]. Larger ICLs, especially the 13.7 mm size had been associated with higher vaults [24], potentially due to a higher effect of the compression forces on the ICL [9]. In the present study, the limited number of eyes implanted with 12.6 and 13.7 mm ICLs may have restricted the ability for detecting the influence of ICL size on the vault. Nonetheless, the inter-eye comparisons showed no statistical and no clinical difference (repeatability for vault measurement ∼60 µm [38]) between sICL and tICL, with the inter-eye differences similar to those observed in fellow eyes implanted with the same ICL geometry [16]. The current results allow us advancing that the vault prediction for an ICL size is independent of the ICL optical geometry.

Postoperative pupil size and vault showed tenuous positive relationships similar for both ICL geometries, i.e. eyes with larger pupils tended to present higher vaults. A recent study by Gonzalez-Lopez et al. using well controlled lightning conditions showed that the postoperative pupil size remained barely unchanged (slightly larger postoperatively ∼0.11 mm) compared to the preoperative pupil size [39]. Therefore, considering a clinically stable pupil size pre- and post-surgery, the association between vault and postoperative pupil size may be explained by the effect anterior–posterior pressure induced by the iris on the ICL.

This study has some limitations. One of them is its retrospective nature, since a previous protocoled study minimises the occurrence of errors and bias. To counteract the occurrence of errors, all AS-OCT were reassessed by redoing the measurements by a proficient operator, unaware of the ICL implanted. Second, there was lack of a planned randomization process as the first operated eye was always the right eye. In our sample 22 eyes were first implanted with sICL and 19 with tICL, representing a good balance between type of ICL initially implanted. A third limitation regards to the sample size as the GEE sees its performance limited for samples lower than 50 individuals [40]. Additionaly, the number of analysed eyes may have limited the detection of factors known to play a role on the vault magnitude; however the study aimed and was designed to detect the influence of ICL optical geometry on the vault using a matched-pairs design. The associations observed between vault and the independent factors were very consistent in the two ICL groups and unlikely to change significantly for larger sample.

## Conclusions

In conclusion, the present findings indicate that the parameter ICL size minus ATA, ICL power and ICL thickness are the major contributors for the vault and the geometrical differences between a sICL and a tICL seem to play a minor role in the vault. Thus, the selection of the ICL size should follow a hierarchical approach with factors such as the ICL size minus the ATA and the ICL power assuming higher relevance. In borderline cases, where two ICLs sizes would be adequate the rotation stability of the tICL needs to be considered and combined with higher hierarchical factors to select the adequate ICL size.

## Supplementary Information

**Additional file 1: Table S1.** Bivariate correlation analysis between vault, measured in eyes implanted with spherical ICL and toric ICL and independent variables.

**Additional file 2:** Raw Data. **Table S2a.** Demographics (Variables: Gender and Age); **Table S2b. **Preoperative refraction (Variables: Sphere, Cylinder and axis, Distance Corrected Visual Acuity (DCVA) for the Right (OD) and Left eye (OS); **Table S2c.** Postoperative refraction (Variables: Sphere, Cylinder and axis, Uncorrected Visual Acuity (UCVA) for the Right (OD) and Left eye (OS); **Table S2d.** ICL Characteristics for the OD and OS (Variables: Type of ICL implanted per eye, Diameter, Sphere, Cylinder and axis); **Table S2e.** Preoperative Pentacam measurements (Variables: Corneal Diameter, Central Corneal Thickness (CCT), Central Keratometry (Kc), Corneal Astigmatism) for the OD and OS; **Table S2f.** Preoperative Visante measurements (Variables: Anterior Chamber Depth (ACD); Anterior Chamber Width (A2A); Central Corneal Thickness (CCT); Crystalline Lens Rise (CLR); Anterior Chamber Angle Nasal and Temporal (ACA Nasal and Temporal) and Pupil diameter) for the OD and OS; **Table S2g.** Postoperative Visante measurements (Variables: ICL Thickness; Vault and Pupil Diameter) for the OD and OS.

## Data Availability

The dataset supporting the conclusions of this article is included within the article and its additional file.

## Abbreviations

ACD: Anterior Chamber Depth
ATA: Horizontal Anterior Chamber Distance
AS-OCT: Anterior Segment Optical Coherence Tomography
CCT: Central Corneal Thickness
CDVA: Corrected Distance Visual Acuity
CLR: Crystalline Lens Rise
GEE: Generalised Estimating Equations
ICL: Implantable Collamer Lens
SE: Spherical Equivalent
sICL: Spherical Implantable Collamer Lens
Sim K: Simulated Keratometry
tICL: Toric Implantable Collamer Lens
UCVA: Uncorrected Distance Visual Acuity
WTW: White to White
