# Supplementary material for: Vault differences in eyes implanted with spherical and toric implantable collamer lenses: an inter-eye analysis
Source: BMC Ophthalmol. 2022 Nov 15;22:435. doi: 10.1186/s12886-022-02653-y (PMC9664806; doi:10.1186/s12886-022-02653-y)
Supplement: Supplementary file 1 — Additional file 1: Table S1. Bivariate correlation analysis between vault, measured in eyes implanted with spherical ICL and toric ICL and independent variables. [file 12886_2022_2653_MOESM1_ESM.docx]

**Bivariate Correlation analysis**

**Table S1.** Bivariate correlation analysis between vault, measured in eyes implanted with spherical ICL and toric ICL and independent variables.

| **Vault**  **Correlation with** | **Spherical ICL**  (Correlation coefficient, R;  p-value) | **Toric ICL**  (Correlation coefficient, R;  p-value) | **Difference in slope magnitude**  p-value |
| --- | --- | --- | --- |
| Age | -0.31; 0.046 * | -0.29; 0.066 | 0.855 |
| ATA | -0.18; 0.250 | -0.30; 0.060 | 0.809 |
| WTW | 0.01; 0.931 | -0.24; 0.128 | 0.906 |
| ICL size – ATA | 0.36; 0.021 * | 0.36; 0.020 * | 0.937 |
| ICL size- WTW | 0.11; 0.495 | -0.40; 0.803 | 0.503 |
| ACQ | 0.09; 0.592 | 0.04; 0.792 | 0.834 |
| CLR | -0.31; 0.052 | -0.24; 0.128 | 0.686 |
| CCT | 0.02; 0.903 | 0.10; 0.533 | 0.768 |
| Sim K | -0.07; 0.659 | 0.11; 0.479 | 0.420 |
| ICL Sphere | -0.08; 0.602 | -0.07; 0.675 | 0.872 |
| ICL Cylinder | - | -0.05; 0.741 | - |
| ICL J0 Cylinder vector | - | -0.26; 0.100 | - |
| ICL J45 Cylinder vector | - | 0.17; 0.279 | - |
| ICL thickness | 0.35; 0.023* | 0.38; 0.014* | 0.839 |
| Pupil (postoperative) | 0.09; 0.570 | 0.05; 0.773 | 0.808 |

*Statistical significance p≤0.05
